# Surgery

**Published:** 1894-08

**Authors:** 


### *SELECTIONS AND ABSTRACTS.*

---

### SURGERY.

EDITED BY DR. F. W. MCRAE.

### GUNSHOT WOUNDS OF THE HEAD.

The French Surgical Congress has devoted considerable time lately to the discussion of this topic with the result of bringing out two diametrically opposing views.

Quénu emphasized the importance of early trephining before symptoms of meningo-encephalitis begin. He claimed that the projectile and the foreign bodies carried into the cranial cavity by it—that is, pieces of the scalp with hair attached, and spiculæ of bone, and fragments of clothing—were sufficient to produce infection *per se*, and consequently they should be removed as soon as possible. He supported his views by relating three cases: 1. A child eight years of age who had a small wound in the frontal region, which had been followed by stupor and suppuration. Three weeks later two fragments of a small bullet were found, after trephining, imbedded in the wound and giving rise to an abscess. At first, following drainage and cleaning out of the cavity, there was improvement in the symptoms, but he soon became worse again; meningo-encephalitis intervened, and the patient died. 2. A man who fired five bullets into his head. Forty-eight hours later, in spite of the application of an antiseptic dressing immediately after the accident, the discharge from the wounds was suspicious; he was trephined on the third day and all the bullets removed. Two were found imbedded in the temporal bone, two were beneath the dura, and the other had perforated the dura. 3. A child of three years was shot in the head by a ball from a Flobert rifle. The dura was not perforated, but some hair was found in the wound in the skull. The wound was carefully cleansed and recovery was uninterrupted. Quénu believes that it is wise to operate at once, and explore the entire wound, for trephining is "inoffensive" when the brain is not included in the operative manipulation. "Not to operate would be imprudent on account of the dangers of later accidents." When the projectile has only penetrated a short distance into the brain substance an attempt should be made to remove it, but when it is deeply situated it is best not to make a dangerous search to locate it. "If the ball is aseptic, intervention is harmless; if it is not aseptic, intervention is indispensable." Despres deprecated searching for a ball that was buried in the brain substance, and Delorme related the results of some experimental work on the cadaver where he found that numerous tracks were made by the spiculæ, which were traversed by the probe, rendering the probability of making a false passage more likely than the finding of the projectile. He says, however, that it is necessary to note the force with which the ball strikes the skull. If the force has been great, which one can determine by inspecting the point of entrance, for in this case it will be small and comparatively smooth, then exploration is out of the question on account of the depth to which the ball has penetrated; but, on the other hand, when the force is slight, exploration should be the rule, for it is probable that the splinters of bone and the ball are all superficially situated.

Reclus depended upon clinical manifestations in determining when to operate, and Monod considered that properly conducted operative methods added no increased dangers, and often, by the removal of the foreign body and the splinters, improved the chances of permanent improvement.

The general tendency in this country is to follow the line of argument laid down by Quénu. The advances in aseptic surgery, and the clever work done in cerebral exploration by Horsley, Keen, Weir and others has led to a decided revolution in the methods of treatment. The brain and its envelopes are no longer dreaded by the surgeon. Roberts has claimed, perhaps with some exaggeration, still with much force, that trephining, *per se*, was no more dangerous than amputation through one of the phalanges, and the statistics now strongly favor the operative rather than the let-alone method of treatment. Wharton's collection of 316 cases of foreign bodies in the brain shows that, even including the cases operated upon prior to antisepsis, sixty-eight per cent. recovered after removal, while in 210 cases where the operation was not done, only forty-one and nine-tenths of one per cent. recovered, and of these several cases had subsequent trouble traceable to the irritation caused by the presence of the foreign body.

Fluhrer's aluminium probe has done much to simplify the search for missiles lodged in the brain substance. It can be allowed to follow the tract simply by force of gravity, and yet it gives distinct evidence of the presence of the ball when it comes in contact with it. It then furnishes plain evidence of the depth of the wound and its direction, and establishes at once the advisability of a counter opening and its position. Girdner's telephonic probe, too, has had considerable influence in determining the procedure in these cases.

The general course of treatment now adopted and advised by surgeons who have had experience with this class of injuries is: To first render the scalp thoroughly aseptic by shaving and washing; then to enlarge the opening either with the trephine or mallet and chisel, removing the detached splinters and withdrawing those that have penetrated the cerebral substance. Then, if the ball has entered the brain, placing the head in such a position that the aluminium probe will follow its track by the force of gravity, it may be traced to its point of lodgment. If it has passed clear through a new opening in the skull at the point where the probe indicates that the ball has probably emerged from the brain, then a new exploration carried on in the same way from the opening in the direcrection of the deflection will enable the operator to locate and remove it. The wounds must then be thoroughly cleansed, drained and sutured, and care taken to avoid hernia cerebri.

---

### "OPERATIVE TREATMENT OF SUCH HERNIÆ AS APPEAR THE RESULT OF CONGENITAL DEFECT OF THE LINEA ALBA, OR AS THE OUTCOME OF LAPAROTOMY."

Dr. J. S. M'Ardle reports following cases. (*Dublin Journal of Medical Science*):

1. Mrs. H., aged fifty-one, came under observation on January 12,

1890. For many years she had noticed a small nodule under the skin, a little above the umbilicus, which, shortly before admission, whilst she was lifting a weight, became suddenly larger and very painful. On examination, a rounded tumor, dull on percussion, irregular in outline, and not reducible, was found a short distance above the umbilicus. Operation was decided upon, and, on opening the sac, a large mass of extremely dark-colored and almost gangrenous omentum, with a knuckle of small intestine, was exposed. The intestine was reduced and the omentum removed. The patient made a good and rapid recovery. Probably the cause of the hernia in this case was weakening of the linea alba by protrusion of a fatty nodule, the removal of which, with suture of the opening, would in all probability have prevented the development of the more serious condition.

2. This was an example of umbilical hernia with strangulated gut and gangrenous omentum. The patient came under observation on March 8, 1891, the hernia being then as large as a fetal head. Operation was at once performed, the strangulated gut being returned to the abdomen and the gangrenous omentum removed. The patient made a good recovery.

3. This was a case of ventral hernia following laparotomy. The hernia was large, and was complicated by ascites. Operation was performed on November 14, 1893. The sac was separated and, after ligature of its neck, was removed. During the operation about half a gallon of clear fluid was removed from the peritoneal cavity. The wound healed by first intention, and up to the present time the progress of the case has been very satisfactory.

In all the above cases the hernia was traceable to weakness of the middle layer of the abdominal wall. In the first case the weakness was due to protrusion of a nodule of fat from the subperitoneal tissue, in the second there was a congenital defect of the middle stratum, and in the third this stratum had failed to unite after laparotomy. Failure of union of the muscular aponeurosis after laparotomy may be due to one of four causes, the order of importance being as follows: (1) Failure to engage the different layers of this stratum sufficiently in the sutures; (2) interposition of contused peritoneum; (3) excessive formation of blood-clot between the layers from defective suturing; and (4) suppuration during the healing of the wound. The author recommends the following method of suturing, which he carried out successfully in the above cases. After freeing the sac a suture is carried round its neck in the subperitoneal tissue and tied. The muscular aponeurosis is next brought together by numerous sutures, and finally the skin is united by sutures which pass down and take up a portion of the aponeurosis.

The paper concludes by referring to some discrepancies in the details of the operation as it is described in recent text-books.—*J. E. Platt, in the Medical Chronicle.*

---

### CORRECTION OF RACHITIC DEFORMITIES AND OF THE DEFORMITIES FOLLOWING INFANTILE AND SPASTIC PARALYSES.*

*I. Correction of Rachitic Deformities.*—The correction of simple out-bowing of the legs in rachitic children before the bones are solidified is easily made by pressure from a properly-applied apparatus. The long out-bowing accompanied by out-knee can also be remedied by mechanical measures. In a few instances where the parties cannot or will not attend to the adjustment of appliances, forcible manual straightening of the tibia and fibula over a fulcrum is advisable, even if a simple or a green-stick fracture is produced.

The epiphyseal line should be carefully guarded against injury when the osteoclast is used. Osteotomy is frequently necessary in extreme cases of out-bowing, especially in large children or in adults.

Anterior curvature of the tibia and fibula rarely yields to apparatus. Manual fracture over a hard fulcrum, or osteoclasis, or preferably osteotomy is advisable. Wedge-shaped osteotomy is seldom necessary except in extreme cases, as even a wide intervening posterior gap can be filled up by callus, and the cuneiform operation is more liable to be followed by suppuration.

---

*Abstracts of papers read before the American Orthopedic Association, at Washington, D. C., May 31, 1894.

For in-knee or out-knee, osteoclasis is inadvisable; but osteotomy, especially above the condyle of the femur, is safe and satisfactory, and is usually sufficient to remove the deformity, except where there is great angularity, in which case a second osteotomy may be performed below the tibial tubercle.

Curvature of the femur, unless it is great, need not be operated upon, as it interferes but little with the walking powers, and is simply a matter of appearance.

*II. Treatment of Deformities following Infantile and Spastic Paralyses.*—1. Prevention of deformity by means of apparatus and other measures is exceedingly important.

2. When contractures have occurred, the employment of surgical measures should precede the application of mechanical measures. To attempt to overcome these deformities by mechanical means alone is to needlessly inflict great pain. It consumes much time, and the final result is no better than by the speedier and less troublesome plan.

3. Myotomy and tenotomy, either subcutaneous or open, are perfectly safe operations. They are usually remarkably effective, and the muscles are placed in a better condition for action than they were before the operation.

4. In contractures at the hip, free open division is usually preferable; at the knee, subcutaneous section can frequently be performed; but when fascial contractions occurring in the central popliteal region are to be overcome, open incision is necessary. At the foot, subcutaneous division is usually sufficient. Tarsectomy is seldom necessary. Fasciotomy is frequently required.

5. Forcible straightening following division is usually necessary. Rectification should be complete at the time of operation, and all contracted tissues should be divided.

6. In spastic paralysis, lengthening of the tendons by tenotomy assists in restoring muscular equilibrium, and consequently secures better subsequent locomotion.

7. Free section of the adductors is often necessary.

8. The best subsequent dressing for maintaining abduction while the patient is in bed is the application of a rigid gypsum dressing to the knee, the legs being then fastened in a widely-separated position.

9. Subsequent to operation for either form of paralysis, mechanical appliances with lock- or stop-joints are frequently temporarily needed, and artificial support by wheeled crutch or other measures will assist in restoring muscular power. Locomotion is the best form of gymnastics.—*DeForest Willard, M.D., Clinical Professor of Orthopedic Surgery in the University of Pennsylvania, in University Medical Magazine.*

---

### A NEW METHOD OF USING COCAINE FOR LOCAL ANESTHESIA.

Krogius describes a new method of producing cocaine analgesia, which is based on the fact that when a solution of this agent is injected into the subcutaneous tissue near to a nerve trunk, it causes loss of sensation over a large zone corresponding to the peripheral distribution of this nerve. In order to reach the selected nerve trunk with certainty, and to apply the cocaine to several of its branches at the same time, the author, in injecting the subcutaneous tissue, passes his needle across the long axis of the limb, and, as the needle is thrust along, the solution is gradually discharged. An injection made in this way across the root of a finger will, in the course of ten minutes, result in analgesia of the whole digit, not of the skin only, but also of the tendons, the periosteum and all the deep structures. If one or two injections be made transversely near the wrist a considerable extent of the palm of the hand may be thus rendered analgesic. The sensibility of the ulnar side of the hand as far as the roots of the last two fingers may, it is stated, be abolished by injecting a solution of cocaine over the ulnar nerve at the back of the elbow. By injecting over both supraorbital notches, analgesia may be produced in the whole of the middle portion of the forhead. The analgesia caused by this method of using cocaine attains its greatest intensity and extent from five to ten minutes after the injection, and is maintained for a quarter of an hour or even longer. The author injects only weak (2 per cent.) solution of cocaine, and keeps the patient recumbent for at least a quarter of an hour after the operation. This method has been practiced with success at Helsingfors in two hundred minor operations, such as amputation of the fingers and toes, excision of palmar fascia and phimosis.—*Centralbl. f. Chir.*—*Times and Register.*

---

### AN ANALYSIS OF TWENTY-EIGHT CASES OF INTUSSUSCEPTION.

Eccles (*Centralblatt für Chirurgie,* No. 32, 1893) says of the twenty-eight reported cases seventeen were males and eleven females. Eighteen were in children under one year, four between one and two years, and three from two to five years, one of seven, one of nine, and one of thirty-nine. The three cardinal symptoms are: Abdominal pain, vomiting and bloody stools. The peculiar form of the abdomen could be made out in eighteen cases.

The treatment may be expectant, mechanical or operative. In four cases the expectant plan was adopted, resulting in two deaths and two recoveries. One case was treated with injections of air and required a secondary laparotomy. Nine were treated with injections of oil, water and milk, resulting in four deaths and five recoveries.—*University Medical Magazine.*

---

### FINDING THE UPPER END OF A DIVIDED TENDON.

Félizet (*Bulletin et Mémoires de la Société de Chirurgie,* 1893, p. 610), in cases in which the tendons are divided, whether at the wrist or hand, advises that the two adjoining fingers be fully extended. This will bring the upper end of the divided tendon into view, and thus enable it to be seized. The affected finger is then flexed, and thus the lower end of the divided tendon is brought up and the two ends are sutured with catgut. In order to prevent too much strain on the catgut sutures, the upper end of the divided tendon, a full centimeter above the point of division, is sewed fast with catgut to the adjoining healthy tendon. In treating the case afterwards the unaffected fingers are kept in a state of extension, while the affected finger is kept in a state of flexion. This position is to be maintained by means of a plaster dressing.

